# Green, Hydrothermal Synthesis of Fluorescent Carbon Nanodots from Gardenia, Enabling the Detection of Metronidazole in Pharmaceuticals and Rabbit Plasma

**DOI:** 10.3390/s18040964

**Published:** 2018-03-24

**Authors:** Xiupei Yang, Mingxian Liu, Yanru Yin, Fenglin Tang, Hua Xu, Xiangjun Liao

**Affiliations:** 1 College of Chemistry and Chemical Engineering, Chemical Synthesis and Pollution Control Key Laboratory of Sichuan Province, China West Normal University, Nanchong 637000, China; mxliu_chem@163.com (M.L.); yyr_anal@163.com (Y.Y.); tfl06180205@126.com (F.T.); bigtree.xu@foxmail.com (H.X.); 2 Exposure and Biomonitoring Division, Health Canada, 50 Colombine Driveway, Ottawa, ON K1A 0K9, Canada; xiangjun.liao@mail.mcgill.ca

**Keywords:** fluorescent carbon nanodots, metronidazole, fluorescent quenching, biological analysis

## Abstract

Strong fluorescent carbon nanodots (FCNs) were synthesized with a green approach using gardenia as a carbon source through a one-step hydrothermal method. FCNs were characterized by their UV-vis absorption spectra, photoluminescence (PL), Fourier transform infrared spectroscopy (FTIR) as well as X-ray photoelectron spectroscopy (XPS). We further explored the use of as-synthesized FCNs as an effective probe for the detection of metronidazole (MNZ), which is based on MNZ-induced fluorescence quenching of FCNs. The proposed method displayed a wide linear range from 0.8 to 225.0 µM with a correlation coefficient of 0.9992 and a limit of detection as low as 279 nM. It was successfully applied to the determination of MNZ in commercial tablets and rabbit plasma with excellent sensitivity and selectivity, which indicates its potential applications in clinical analysis and biologically related studies.

## 1. Introduction

Metronidazole (MNZ) is a derivative of nitroimidazole and commonly used to treat human diseases, including parasitic infection, trichomoniasis, giardiasis and amebiasis [1,2]. MNZ has also been employed as a veterinary medicine to prevent and treat infections or to promote growth and improve feed conversion efficiency [3]. However, when the accumulated dose of MNZ exceeds the therapeutic threshold for humans, some toxic effects will be caused. For instance, seizures, peripheral neuropathy and ataxia [4]. For this reason, MNZ, along with several other nitroimidazoles, has already been banned in Europe. The uncontrolled use of MNZ or accidentally distributing feeds contaminated by MNZ, may result in its residues being present in edible tissues. Therefore, it is of great importance to accurately detect the content of MNZ in pharmaceuticals and biological samples. A variety of quantitative analytical strategies has been reported for the detection of MNZ, which mainly include high performance liquid chromatography (HPLC) [5], gas chromatography (GC) [6], thin layer chromatography (TLC) [7], spectrophotometry [8] and electrochemical sensors [9,10,11]. Considering some drawbacks of those methods, such as the time consuming sample preparation and sophisticated instrumentation required, the need for a better analytical method remains a challenge. Apart from the aforementioned methods, fluorescence analysis has attracted considerable attention due to its relatively low cost, high sensitivity, ease of operation, reliable approach and its low detection limit [12]. Recently, fluorescent carbon nanodots (FCNs), a new class of carbon nanomaterials, have been actively researched and well applied in biosensors, biomarkers, biomedical imaging and so on [13,14,15,16,17]. In striking contrast to semiconductor quantum dots, FCNs are superior fluorescent nanomaterials due to their low toxicity, high chemical stability and low environmental impact. There are many examples in the literature of using some plants [18,19], polysaccharides [20] or other organic molecules [21] for the synthesis of FCNs. In 2015, we developed a novel method for the preparation of FCNs by hydrothermal treatment of aloe. By employing the new FCNs, we successfully demonstrated a sensitive sensor for the detection of tartrazine in food samples [22]. Recently, Hatamie et al. used graphitic carbon nitride as a fluorescent carbon material for detecting MNZ [23]. 

*Gardenia jasminoides* (Ellis.) is widely cultivated in Asia, especially in the south of China. It is a genus of about 250 species of flowering plants in the family Rubiaceae, which is native to the tropical and subtropical regions of Africa, Southern Asia, Australasia and Oceania. In traditional Chinese medicine (TCM), Fructus Gardenia, the dried ripe fruit of *Gardenia jasminoides* (Ellis.), is used to cure patients with inflammation, viral encephalitis, hepatitis, tonsillitis, tracheitis and high fever. Here, we hope that gardenia flowers can also produce similar FCNs that can be used as fluorescent probes. To the best of our knowledge, there have been no reports of the use of gardenia as a carbon source for the synthesis of FCNs or for their use as ametronidazole fluorescent probe.

In this work, we demonstrated a simple, low cost, and green preparative strategy toward water-soluble nitrogen-doped FCNs by hydrothermal treatment of gardenia at 220 °C. The carbonization, surface functionalization and doping occurred simultaneously during the hydrothermal treatment, which led to the formation of the nitrogen-doped FCNs. Moreover, the fluorescence of FCNs gradually decreased with an increase in MNZ concentration. Herein, we report the development of a fluorescence sensing probe based on FCNs for the detection of trace amounts of MNZ. The analytical feature and the application of the proposed fluorescence quenching method have been fully explored. The major attributes of the proposed method are its simplicity, cost-effectiveness, convenience and environmental friendliness.

## 2. Experimental

### 2.1. Materials

Metronidazole, ronidazole, secnidazole and dichloremethane were purchased from Shanghai Aladdin Co. (Shanghai, China). Sodium dihydrogen phosphate (NaH_2_PO_4_·2H_2_O) and disodium hydrogen phosphate dodecahydrate (Na_2_HPO_4_·12H_2_O) were supplied by Tianjin Fuchen Chemical Reagents Co., Ltd. (Tianjin, China). Two kinds of MNZ tablets were purchased at local drug stores. Reagents and materials, such as glucose, sucrose, starch, β-cyclodextrin, ascorbic acid, urea, NH_4_Cl, NaCl, KNO_3_, MgSO_4_, NaCO_3_ and amino acids were used as received without further purification. The gardenias were obtained from the school yard of China West Normal University and washed by water for further use. The ultrapure water used throughout the experiments was purified through a UPH-II-20 Tap water purification system (Chengdu Ultrapure Technology Co., Ltd., Chengdu, China).

### 2.2. Apparatus

The FCNs were synthesized in a 100 mL hydrothermal autoclave (Weihai Huixin Chemical Machinery Co., Ltd.). All absorption spectra were recorded on a Shimadzu UV-2550 UV-vis absorption spectrophotometer (Kyoto, Japan). Fluorescence measurements were conducted with a Cary Eclipse fluorescence spectrophotometer (Varian, Palo Alto, CA, USA). The infrared spectra were obtained on a Nicolet 6700 Fourier transform infrared (FTIR) spectrometer (Thermo Electron Corporation, Waltham, MA, USA) with passed KBr pellet at room temperature. Transmission electron microscope (TEM) measurements were carried out on a JEM-1200EX (JEOL, Tokyo, Japan) with an acceleration voltage of 250V. X-ray photoelectron spectroscopic (XPS) measurements were carried out on an ESCALAB 250Xi (Waltham, MA, USA) and the equipped with PF4 (peek fit 4) software was used to deconvolute the narrow scan XPS spectra.

### 2.3. Preparation of FCNs

The FCNs were synthesized from gardenias through a simple, convenient and one-step hydrothermal method. Briefly, the fresh gardenia flowers were first washed in water and dried in sunlight, followed by oven-drying at 100 °C for 10 h to allow for carbonization. Then, 2.0 g of the pretreated gardenias was added into 50 mL of H_2_O. The resulting mixture was transferred into a 100-mL Teflon-lined autoclave and kept at 180 °C for 6 h. After the autoclave cooled down naturally, the obtained brown-yellow solution was filtrated with a 0.22 μm membrane and the FCNs were collected by removing the unreacted organic moieties through treatment with dichloromethane. Finally, the light yellow aqueous solution containing FCNs was collected and diluted 13.3 times with ultrapure water, then stored at 4 °C for further characterization and use.

### 2.4. Quantum Yield Measurements

The quantum yield of the as-synthesized FCNs was measured on the basis of a procedure described previously. Quinine sulfate in 0.1 M H_2_SO_4_ was used as a reference standard, for which the quantum yield was 0.54 at 360 nm reported by the literature. Absolute values of the quantum yield were calculated according to the equation

$$\Phi_{x}{=\Phi }_{\mathrm{std}}\frac{I_{x}}{A_{x}}\frac{A_{\mathrm{std}}}{I_{std}}\frac{\eta_{x}^{2}}{\eta_{std}^{2}},$$

where Φ is the quantum yield of the as-symmetrical FCNs, *A* is the absorbance, *I* is the corrected emission intensity at the excitation wavelength, and *η* is the refractive index of the solvent. The subscripts “*std*” and “*x*” refer to a reference standard with known quantum yield and the FCNs solution, respectively. For the sake of reducing the effects of reabsorption within the sample on the observed emission spectrum, the absorbance values (*A*) of all solutions in the 10 mm cuvette were always controlled under 0.1.

### 2.5. Preparation of Samples

All tablet samples (Metronidazole Tablets, MNZ declared of 200 mg/tablet, Huanan Pharmaceutical Group Co., Ltd., Guangdong, China) were purchased in the local market. Twenty tablets were finely powdered and the equivalent of one tablet (200 mg as MNZ) was accurately weighed and extracted with 100 mL of water. It was sonicated at room temperature for 5 min. The solution was filtered through an ordinary filter paper, washed with water several times and the filtrate plus washings were diluted to the mark in a 250 mL calibrated flask. The sample solutions were diluted with water to obtain solutions where the expected concentration of MNZ was within the calibration range. 

An oral administration of 100 mg of MNZ was given to healthy rabbits (1.8–2.5 kg), rabbit plasma samples (2–3 mL) were collected from the veins of rabbit ears at 5.0 h after dosing. The samples were kept in an ice-bath until being centrifuged at 5000× *g* rpm for 15 min at 4 °C. The serum was carefully collected and stored at −80 °C. Before assay, 0.50 mL of serum sample was diluted with 0.50 mL of 0.60 M trichloroacetic acid aqueous solution and shaken vigorously for 15 min to deposit proteins. It was then left at 0 °C for 1 h. After further centrifuging at 10,000× *g* rpm for 15 min, the supernatant was used as the sample in the subsequent experiments.

### 2.6. Detection of MNZ

A stock solution of MNZ (10.0 mM) was prepared in water. Working solutions were acquired by serial dilution of the stock solutions. For the determination of MNZ, 500 μL of FCNs, 1000 μL of phosphate buffer solution (PBS, pH = 7.0) and an appropriate volume of MNZ stock solution or sample solution were successively added into a 5.0 mL brown centrifuge tube. The mixture was diluted to 4000 μL with water and incubated at 30 °C for 10 min prior to fluorescence measurement. The fluorescence spectra were recorded under excitation at 355 nm and the excitation and emission slits were set at 10 nm. The sensitivity and selectivity measurements were conducted in sextuplicate. All recoveries were calculated based on the equation below:

$$Recovery=\left(C_{measured}-\;C_{initial}\right)/C_{added},$$

where *C_measured_* is the measured sample concentration after spiking, *C_initial_* is the measured sample background concentration and *C_added_* is the known concentration of the spike.

## 3. Results and Discussion

### 3.1. Optimization of the Synthesis

To ensure excellent performance of the synthesized FCNs, optimization of parameters such as the mass of gardenia used, the reaction time and temperature for the synthesis was undertaken and the results are shown in Figures S1–S3. From Figure S1, we can see clearly that the fluorescence intensity of the obtained FCNs solution reached maximum fluorescence intensity with 2.0 g gardenia as a carbon source and 300 μL of the final synthesized solution. As a result, 2.0 g of gardenia and 300 μL of the final synthesized solution were selected for the entire course of the experiment. As can be seen from Figure S2, the fluorescence intensity gradually increased with the reaction time up to 10 h but decreased thereafter. Therefore, 10 h was chosen as the optimal reaction time. As displayed in Figure S3, the fluorescence intensity greatly increased as the reaction temperature was raised from 200 to 220 °C. Only a slight difference in fluorescence intensity was observed between 220 °C and 225 °C. Hence, are action temperature of220 °C was chosen for the subsequent experiments.

### 3.2. Characterization of FCNs

**Figure 1:**
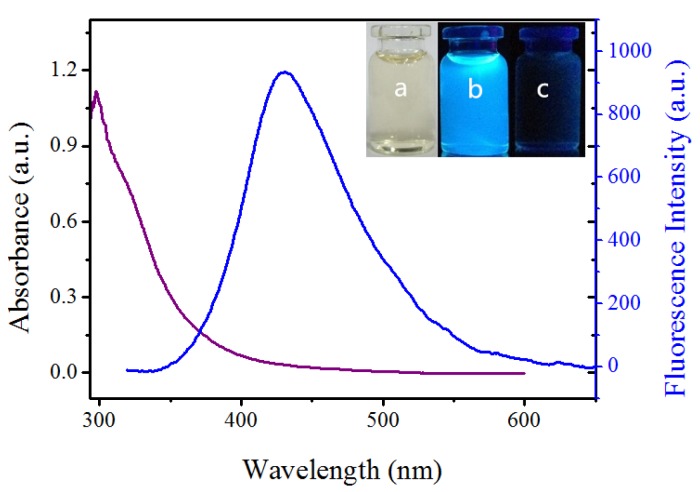
UV-vis absorption (**red**) and fluorescence emission (**blue**) spectra of the fluorescent carbon nanodots (FCNs).

To explore the optical properties of the FCNs, UV-vis absorption spectra and photoluminescence (PL) were studied at room temperature. As shown in Figure 1, there is an absorbance band centered around 298 nm in the UV-vis absorption spectrum. The peak at 298 nm can be ascribed to the π→π* transition of C=C [24]. The PL spectrum shows an optimal emission peak about 431 nm when excited at 355 nm. Accordingly, from the photographs shown in Figure 1 inset, the diluted FCNs aqueous solution is light yellow under daylight (a) but exhibits a very intense blue color when irradiated with a 365 nm UV light (b) further indicating the blue fluorescent property of the FCNs. Fluorescence under 365 nm UV light was quenched after adding MNZ (c). The strong fluorescence of the FCNs may result from the emissive traps of the nitrogen-doped surface [25,26,27]. 

**Figure 2:**
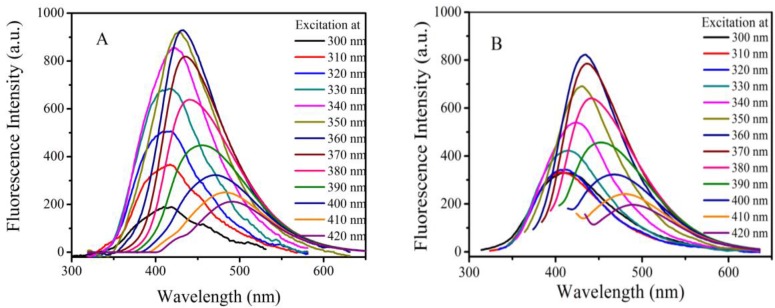
Fluorescence emission spectra of FCNs obtained at different excitation wavelengths without metronidazole (**A**) and adding metronidazole (15 μM) (**B**).

As seen with other carbon-based quantum dots [28,29,30], the fluorescence of the current FCNs also changes with the excitation wavelength. Figure 2A displays the maximum PL peak shifts from 415 to 496 nm with a change in excitation wavelength from 300 to 420 nm. Such excitation-dependent PL behavior is related to the different surface states of the nitrogen-doped carbon nanodots [31]. After the addition of metronidazole, as shown in Figure 2B, the fluorescence intensity of the FCNs was reduced. However, the trend of the position of the fluorescence emission peak is basically the same as in Figure 2A.The results showed that after the addition of metronidazole, the fluorescence excitation-dependent properties of FCNs did not change significantly.

**Figure 3:**
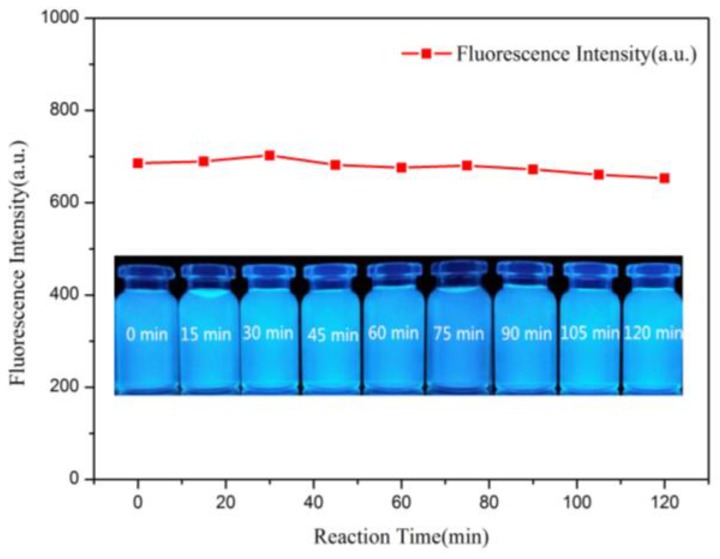
Photostability study of FCNs under UV light irradiation.

To improve the stability of FCNs for detection and analysis, the fluorescence stability of the synthesized FCN solution was explored and the results are shown in Figure 3. Even after being irradiated with a UV lamp at 365 nm for 2 h and after the stability was confirmed by fluorescence spectroscopy, no bleaching was clearly observed under UV light irradiation, indicating that FCNs have good photo stability. Using quinine sulfate as a reference, a PL quantum yield (QY) of 9.82% was measured. 

**Figure 4:**
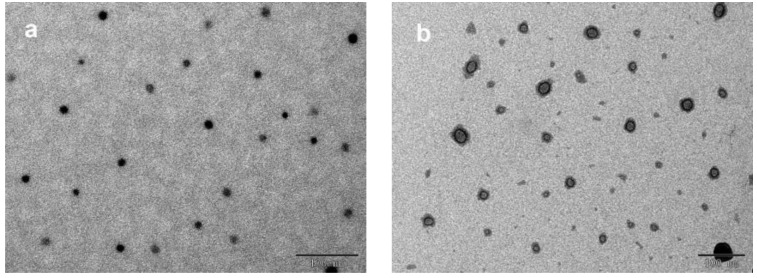
Transmission electron microscope (TEM) images of FCNs in the absence of metronidazole (MNZ) (**a**) and presence of MNZ (**b**).

Figure 4 shows the typical TEM images of the as-synthesized FCN solution in the absence and presence of MNZ (125.00 μM). It revealed that the FCNs were well dispersed with regular spherical shape and had an average size of 9 nm approximately. However, when metronidazole was added, the morphology of the FCNs changed greatly, the size of the nanoparticles became extremely irregular, the surface of the particles became blurred and particle agglomeration was clearly observed.

**Figure 5:**
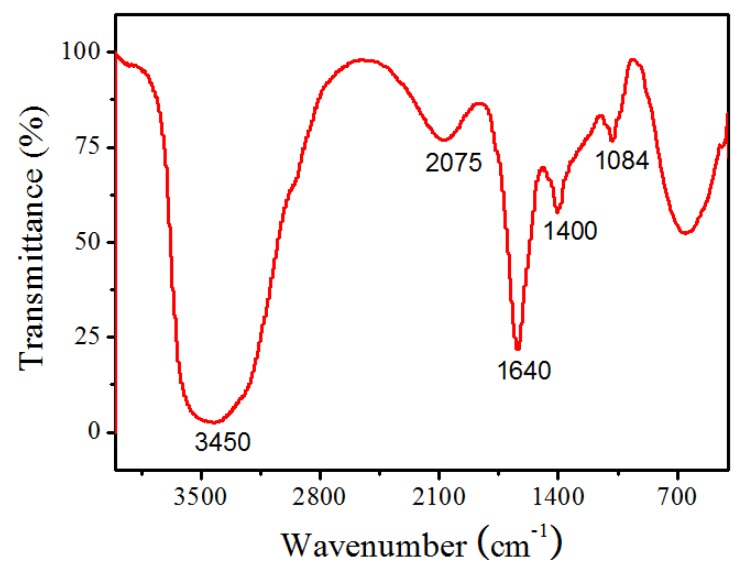
Fourier Transform Infrared spectra of the FCNs.

The FTIR spectrum in Figure 5 shows that multiple functional groups are present on the surface of FCNs, including the stretching vibration characteristic absorption peaks from –OH at 3450 cm^−1^ and 1084 cm^−1^, the asymmetric and symmetric stretching vibration peaks of COO– at 1640 cm^−1^ and 1400 cm^−1^, respectively [32].

**Figure 6:**
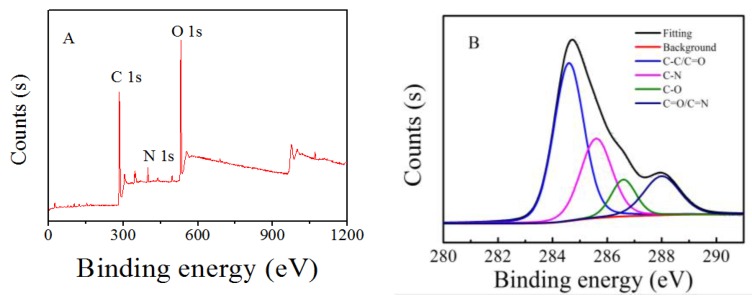

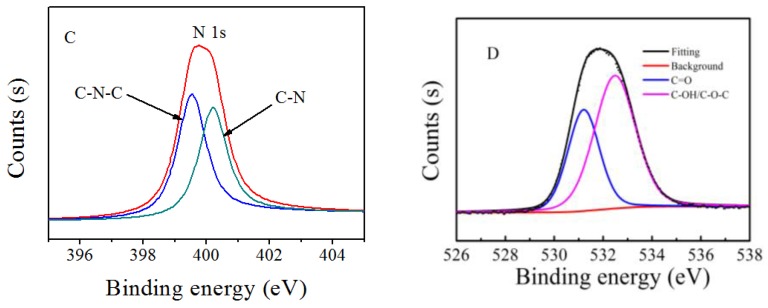
Survey X-ray photoelectron spectroscopy (XPS) spectra of FCNs (**A**) and high-resolution XPS data of C1s (**B**), N1s (**C**) and O1s (**D**).

To further investigate the components, surface groups and the structure of the as-synthesized FCNs, XPS was carried out. The three peaks at 284.30, 398.8 and 532.04 eV shown in the XPS spectrum of these nanoparticles (Figure 6A) can be attributed to C1s, N1s, and O1s, respectively. The XPS results indicate that these nanoparticles are mainly composed of carbon, oxygen, as well as a limited amount of nitrogen. The C1s spectrum (Figure 6B) shows three peaks at 284.3, 285.9 and 288.1 eV, which can be assigned to C–C/C=C, C–OH/C–O–C and C=O/C=N, respectively. The two peaks at 398.9 and 399.8 eV in the N1s spectrum (Figure 6C) come from C–N–C and C–N groups, respectively. The O1s spectrum (Figure 6D) shows two peaks at 532.7 and 531.9 eV, which are due to the existence of C–OH/C–O–C and C=O bands, respectively [33,34]. These results are consistent with those from FTIR.

### 3.3. Principle of the Fluorescence Sensor

**Scheme 1:**
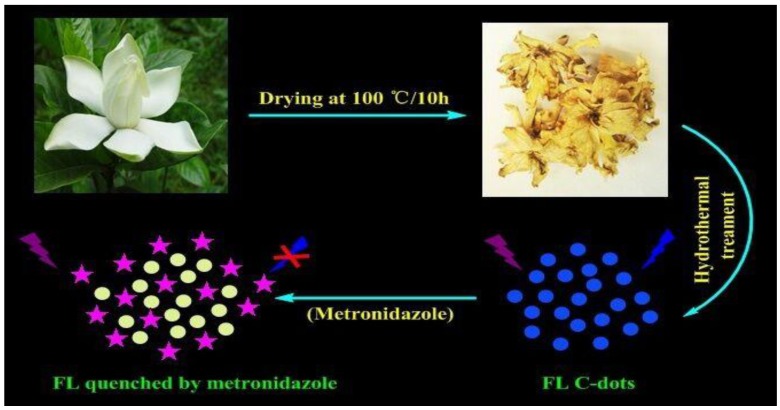
Scheme of the synthetic strategy for FCNs and the principle of metronidazole sensing.

The synthetic strategy for FCNs and the principle of MNZ sensing are represented in Scheme 1. The as-prepared FCNs exhibited strong blue fluorescence. After the addition of MNZ, the fluorescence intensity of the FCNs decreased significantly. These results suggest that the FCNs could be used as a facile fluorescence quenching sensor for MNZ with high sensitivity based on the interaction between MNZ and FCNs.

### 3.4. Mechanism of Fluorescence Quenching

**Figure 7:**
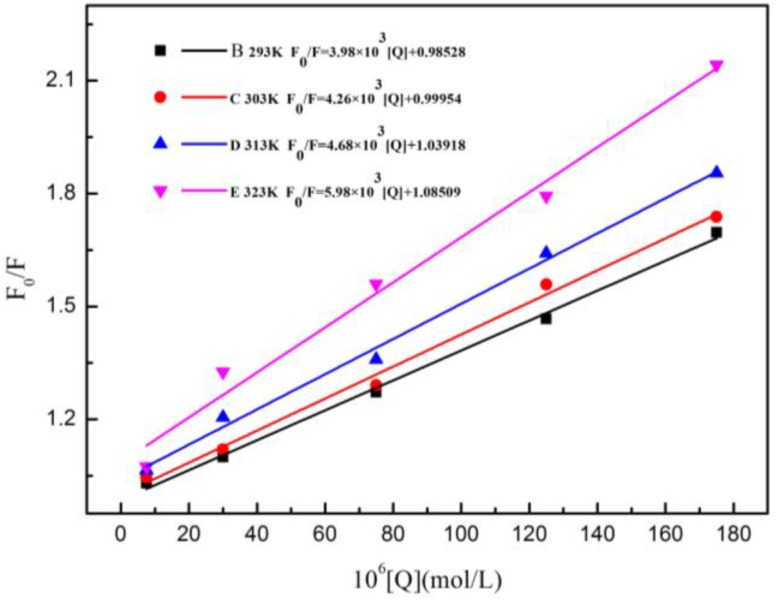
Stern–Volmer plots for the system of FCNs in the presence of metronidazole under temperatures of 293, 303, 313, and 323 K, respectively. F_0_ and Fare the fluorescence intensity of FCNs in the absence and presence of metronidazole, respectively. Conditions: FCNs, 500 μL; PBS, 0.2 M, pH = 7.0.

Broadly speaking, various kinds of molecular interactions with the quencher molecule can reduce the fluorescence quantum yield, such as electron or energy transfer, collisional quenching, excited-state reaction and ground-state complex formation. The quenching mechanisms are usually divided into dynamic quenching (which results from collision) and static quenching (resulting from the formation of a ground-state complex between the fluorescence material and quencher). On the other hand, they could be further distinguished by features such as the relationship between quenching and viscosity, temperature, and lifetime measurements. In general, the dynamic fluorescence quenching constants will increase with arise in the temperature of the system due to changes in the energy transfer efficiency and the increase in effective collision times between molecules. On the contrary, the values of the static fluorescence quenching constants will decrease with a rise in temperature of the system. Let us suppose that the mechanism is dynamic quenching; it can be described by the Stern–Volmer equation.

$$F_{0}/F\;=\;1\;+\;K_{\mathrm{sv}}\left[Q\right]\;=\;1\;+\;K_{q}\tau_{o}\left[Q\right],$$

where F_0_ and F are the FCNs fluorescence intensities at 360 nm in the absence and presence of metronidazole, respectively; K_SV_ and K_q_ are the Stern–Volmer quenching constant and the bimolecular quenching constant, respectively; [Q] is the concentration of metronidazole; and τ_o_ is the average lifetime of the FCNs without any other fluorescence quencher with a general value of 10^−8^ s. Figure 6 shows the fluorescence intensities of the FCNs analyzed by plotting F_0_/F against [Q] at 293, 303, 313, and 323 K. Table 1 summarizes the calculated K_SV_ and K_q_ values for each temperature. As shown in Figure 7, as the temperature increases, the fluorescence quenching constant increases gradually and increasing the temperature favors the fluorescence quenching. This is consistent with the dynamic quenching mechanism. These findings indicate that the quenching process may be caused by dynamic quenching.

### 3.5. Optimal Conditions for FCNs Detection

#### 3.5.1. Effect of pH

**Figure 8:**
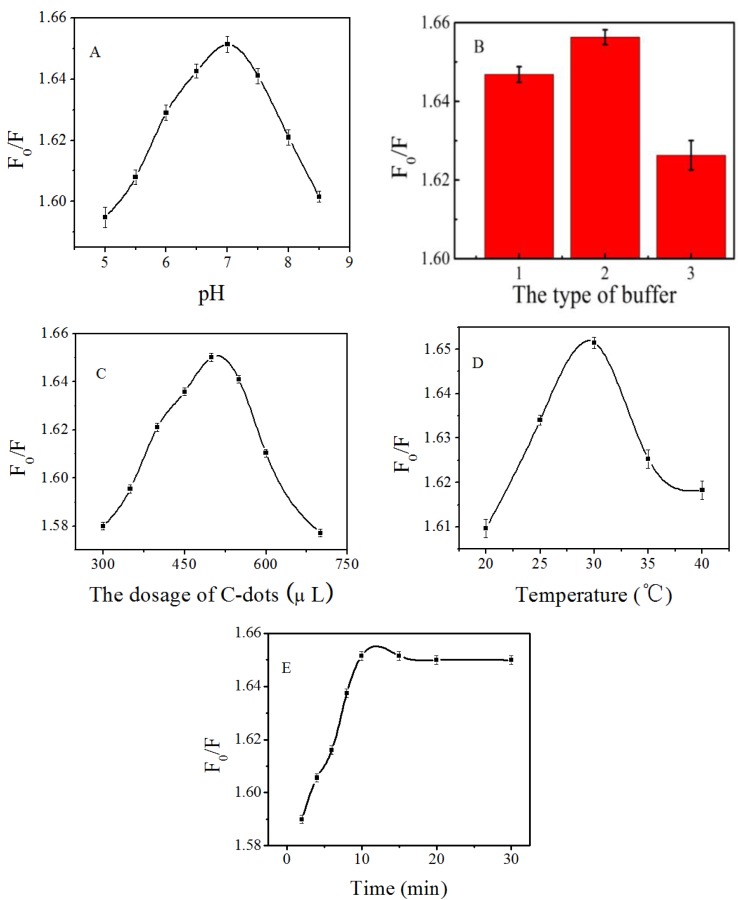
Effect of (**A**) pH; (**B**) the type of buffer (1. potassium phosphate, 2. sodium phosphate and 3. citrate solution); (**C**) the dosage of FCNs; (**D**) reaction temperature and (**E**) reaction time on the fluorescence intensity of the FCNs-MNZ system. Conditions: PBS, 0.2 M; MNZ, 125.00 μM.

The dependence of FCNs fluorescence upon pH in the presence of MNZ is shown in Figure 8A. In this work, the fluorescence quenching efficiency of MNZ on FCNs is defined as F_0_/F, where F_0_ and F are the fluorescence intensities of FCNs in the presence and absence of MNZ, respectively. An increase in pH from 5 to 7 leads to an increase in the fluorescence quenching efficiency of the system, whereas a further increase in pH results in the decrease in F_0_/F. At the same time, the effects of different buffer systems on fluorescence quenching efficiency were compared. As can be seen from Figure 8B, the highest quenching efficiency was obtained from a sodium phosphate buffer system. Therefore, sodium phosphate buffer solution at pH = 7 was selected for the following experiments.

#### 3.5.2. Effect of the Dosage of FCNs

The effect of the FCN dosage on the quantitative detection of MNZ is presented in Figure 8C. The fluorescence quenching efficiency of the system increased gradually with an increase in the FCN dosage from 300 to 500 μL. When the dosage of FCNs was greater than 500 μL, the quenching efficiency decreased. Consequently, a dosage of 500 μL was used for subsequent experiments.

#### 3.5.3. Effect of Reaction Temperature

The effects of the reaction temperature on the fluorescence quenching efficiency of the system were studied at 20, 25, 30, 35, and 40 °C. As shown in Figure 8D, the quenching efficiency increased with an increase in reaction temperature from 20 to 30 °C but decreased thereafter. Hence, 30 °C was selected as the optimum reaction temperature.

#### 3.5.4. Effect of Reaction Time

The effect of reaction time on the fluorescence quenching efficiency of the system is shown in Figure 8E. The fluorescence quenching efficiency of the system gradually increased from 1 to 10 min then no significant changes in F_0_/F were observed after a reaction time of 10 min. Thus, 10 min was considered as the optimum reaction time.

### 3.6. Selectivity of the Proposed Method

**Figure 9:**
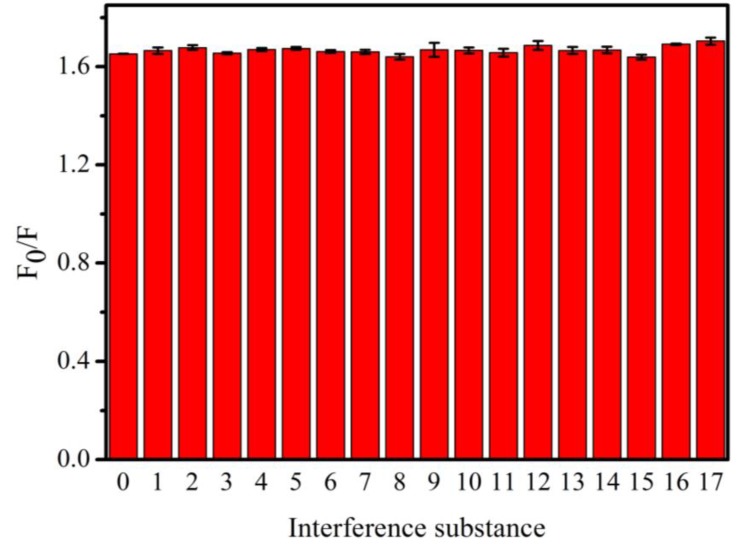
Effects of potentially interfering substances: (0) non-interference; (1) K^+^, 25 mM; (2) Na^+^, 25 mM; (3) Mg^2+^, 25 mM; (4) NH${}_{4}^{+}$, 25 mM; (5) NO${}_{3}^{-}$, 25 mM; (6) SO${}_{4}^{2-}$, 25 mM; (7) CO${}_{3}^{2-}$, 25 mM; (8) glucose, 25 mM; (9) lactose, 25 mM; (10) starch, 25 mM; (11) oxalic acid, 25 mM; (12) glycine, 25 mM; (13) l-histidine, 25 mM; (14) ascorbic acid, 6.25 mM; (15) β-cyclodextrine, 6.25 mM; (16) ronidazole, 625 μM; (17) secnidazole, 625 μM. Conditions: FCNs, 500 μL; PBS, 0.2 M, pH 7.0; MNZ, 125.00 μΜ.

To verify the selectivity of the method, we investigated possible interferences from common inactive ingredients presented in the drug. As shown in Figure 9, when the concentrations of K^+^, Na^+^, Mg^2+^, NH${}_{4}^{+}$, NO${}_{3}^{-}$, SO${}_{4}^{2-}$, CO${}_{3}^{2-}$, glucose, lactose, starch, oxalic acid, glycine and l-histidine were 200 times that of MNZ, no obvious interference was observed. A similar observation was made for ascorbic acid and β-cyclodextrin where their concentrations were 50 times that of MNZ. For ronidazole and secnidazole, however, no interference was observed when the concentrations of the two substances were comparable with the concentration of MNZ. Considering that the quenching of fluorescence can also be caused by other molecules with absorption spectra that overlap with the emission spectra of FCNs, we compared UV absorption spectra between interference, analytes, and FCNs (Figures S4–S6). The results demonstrate that fluorescence quenching due to spectral overlap does not occur under selected experimental conditions. When the concentrations of ronidazole and secnidazole reached five times that of MNZ, the results were compromised. All in all, common inactive ingredients that exist in drugs did not affect the detection of MNZ, which indicates that the method proposed here can be used for the selective detection of metronidazole in drugs. Of course, extra pretreatment steps maybe required to eliminate similar interferences in biological samples.

### 3.7. Fluorescence Detection of MNZ

**Figure 10:**
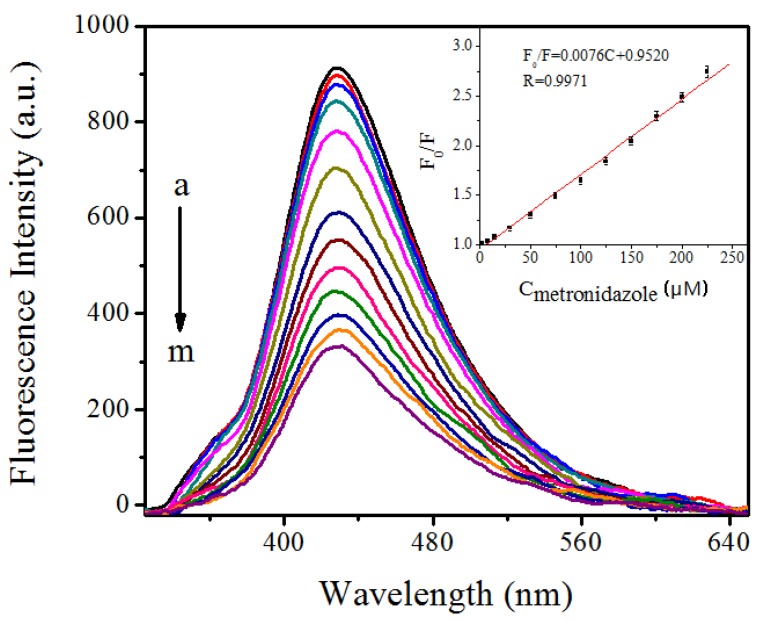
The change in fluorescence intensity of FCNs upon the addition of various concentrations of MNZ. The concentrations of MNZ are as follows: (**a**) 0.00; (**b**) 2.50; (**c**) 7.50; (**d**) 15.00; (**e**) 30.00; (**f**) 50.00; (**g**) 75.00; (**h**) 100.00; (**i**) 125.00; (**j**) 150.00; (**k**) 175.00; (**l**) 200.00; and (**m**) 225.00 μM. Conditions: PBS, 0.2 M, pH = 7.0; FCNs: 500 μL.

Figure 10 shows the change in fluorescence intensity of FCNs upon the addition of various concentrations of MNZ. As displayed, the fluorescence quenching efficiency of FCNs gradually decreased with an increase in the concentration of MNZ. As shown in the upper right inset of Figure 10, the decrease in fluorescence quenching efficiency exhibited a linear response to the MNZ concentration in the range of 0.8–225.0 μM. The calibration curve can be depicted as F_0_/F = 0.0076C+0.9520 (C is the concentration of MNZ, μM) with a correlation coefficient of 0.9971 and with a limit of detection (LOD) at 279 nM. LOD is defined by the equation LOD = 3S_0_/K, where S_0_ is the standard deviation of blank measurements (*n* = 7) and K is the slope of the calibration curve. The repeatability of the proposed method was also evaluated by performing a series of ten for 125.0 μM MNZ and a relative standard deviation (RSD) of 0.72% was obtained. This result suggests that our assay protocol is very precise.

### 3.8. Applications

To test the feasibility of our protocol, the proposed method was used to determine the concentration of MNZ in two different brands of metronidazole tablets and to test the recovery from the spiked tablets. The results are depicted in Table 2. The amount of MNZ measured in the tablets with our method is comparable to that of the method reported in pharmacopoeia [35]. The recoveries of 92.8–104.4% and RSD of 0.44–1.54% were achieved. These results confirm that the proposed sensor provided good precision and accuracy and that it could potentially be used for the detection of MNZ in biological samples. Table 3 presents the results of recovery tests using rabbit plasma. Good recovery (95–105%) and high precision (RSD < 3%) are obtained, indicating the applicability of the developed method in the analysis of biological samples.

### 3.9. Method Comparison

A comparison of method performance between this study and others in terms of sensitivity and linear range is presented in Table 4. Our developed assay exhibits a lower LOD compared to most other studies. It is worth mentioning that our method can be an alternative to others for the determination of MNZ in samples as it avoids the use of sophisticated techniques, complicated operations and the requirement of skilled operators.

## 4. Conclusions

Strong blue-emitting fluorescent carbon nanodots (FCNs) using gardenia as a carbon source were synthesized via a simple and green hydrothermal method and their characterizations are elucidated. Without further chemical modification, the synthesized FCNs have been applied to the detection of metronidazole with high sensitivity and selectivity in commercial tablets and rabbit plasma. Our studies have proved that FCNs could be a useful and powerful luminescence tool for chemical analysis.

## Figures and Tables

**Table 1:** Stern–Volmer quenching constants for the interaction of C-Dots and metronidazole at different temperatures.

pH	T(K)	K_SV_(L·mol^−1^)	K_q_(L·mol^−1^·S^−1^)	R	SD
7.0	293	3.98 × 10^3^	3.98 × 10^11^	0.99858	0.01681
7.0	303	4.26 × 10^3^	4.26 × 10^11^	0.99734	0.0246
7.0	313	4.68 × 10^3^	4.68 × 10^11^	0.99754	0.02601
7.0	323	5.98 × 10^3^	5.98 × 10^11^	0.99321	0.0555

**Table 2:** Recovery and precision of metronidazole in tablets (*n* = 6).

Samples ^1^	Detected (μM)	Spiked (μM)	Found (μM)	Recovery (%)	RSD (%)	Pharmacopoeia Method ^2^ (mg/tablet)
Tablet 1#	22.94 ± 0.23	15	38.39 ± 0.33	103.0	0.44	22.98 ± 0.26
		30	52.61 ± 0.45	98.9	0.50	
		45	65.49 ± 0.96	94.6	1.13	
Tablet 2#	23.95 ± 0.36	15	39.60 ± 0.81	104.4	1.02	23.87 ± 0.40
		30	53.63 ± 1.29	98.9	1.20	
		45	65.72 ± 1.44	92.8	1.54	

^1^ Tablet 1#: batch No. 140602, expiry date: 06/2016, from Jilin Wantong Pharmaceutical Group Zhengzhou Wantong pharmaceutical Co., Ltd., Zhengzhou, China; Tablet 2#: batch No. 150201, expiry date: January 2018, from Great Medicine (China) Co., Ltd., Wuhan, China. ^2^ UV-vis method.

**Table 3:** Metronidazole determination in rabbit plasma (*n* = 6).

Rabbit Plasma	Concentration (μM)			Recovery (%)	RSD (%)
	Initial	Added	Found		
1	11.24 ± 0.42	10	21.06 ± 0.58	98.2	2.20
		20	31.96 ± 0.96	103.6	1.86
		30	39.98 ± 1.07	95.8	2.75
2	9.70 ± 0.38	10	20.17 ± 0.80	104.7	2.41
		20	29.10 ± 1.28	97.0	1.59
		30	38.62 ± 1.50	96.4	2.63

**Table 4:** Comparison of the proposed method with other methods for the determination of metronidazole.

Method	Linear Range (mol L^−1^)	LOD (mol L^−1^)	Ref.
Flow-injection chemiluminescence method	1.46 × 10^−7^–4.38 × 10^−3^	6.31 × 10^−10^	[36]
Electrochemical detection	1 × 10^−9^–2 × 10^−6^	1 × 10^−8^	[37]
Electrochemical method	1 × 10^−7^–2.5 × 10^−5^	4.7 × 10^−8^	[38]
Electrochemical technique	5 × 10^−7^–4 × 10^−4^	3.7 × 10^−7^	[39]
Electrochemical sensor	9.99 × 10^−10^–1.20 × 10^−6^	2.874 × 10^−10^	[40]
Kinetic spectrophotometric H-point standard addition method	2.92 × 10^−5^–1.46 × 10^−4^	4.85 × 10^−6^	[41]
Reversed-phase high performance liquid chromatography	5.84 × 10^−5^–4.09 × 10^−4^	1.93 × 10^−6^	[42]
Electrochemical reduction	2.5 × 10^−8^–1 × 10^−5^	6 × 10^−9^	[43]
High performance liquid chromatography	7.60 × 10^−7^–1.75 × 10^−3^	7.60 × 10^−7^	[44]
Spectrophotometric determination	5.84 × 10^−6^–9.93 × 10^−5^	-	[45]
Electrochemical sensor	6 × 10^−14^–4 × 10^−12^	2 × 10^−14^	[46]
Ion Mobility Spectrometry	2.92 × 10^−7^–4.09 × 10^−4^	2.57 × 10^−8^	[47]
Cathodic stripping voltammetric method	3.30× 10^−10^–4.49 × 10^−6^	2.10 × 10^−10^	[10]
Turn-off FCNs	2.5 × 10^−6^–2.25 × 10^−4^	2.79 × 10^−7^	This work

## Supplementary Materials

The following are available online at http://www.mdpi.com/1424-8220/18/4/964/s1, Figure S1: Effect of various qualities of gardenia for synthesizing FCNs and the differential dilution ratio on the fluorescence intensity of FCNs solution at 220 °C for 10 h; Figure S2: Fluorescence spectra (A) and fluorescence intensity (B) of C-dots prepared under various reaction times; Figure S3: Fluorescence spectra (A) and fluorescence intensity (B) of C-dots prepared under various temperature; Figure S4: Overlapping between Flurescence spetra of FCNs and the UV-vis absorption spectra of ronidazole; Figure S5. Overlapping between Flurescence spetra of FCNs and the UV-vis absorption spectra of secnidazole; Figure S6. Overlapping between Flurescence spetra of FCNs and the UV-vis absorption spectra of glucose, Na^+^ and Mg^2+^; Figure S7. Overlapping between flurescence spetra of FCNs and the UV-vis absorption spectra of metronidazole.
