# Supplementary material for: Green, Hydrothermal Synthesis of Fluorescent Carbon Nanodots from Gardenia, Enabling the Detection of Metronidazole in Pharmaceuticals and Rabbit Plasma
Source: Sensors (Basel). 2018 Mar 24;18(4):964. doi: 10.3390/s18040964 (PMC5949037; doi:10.3390/s18040964)
Supplement: Supplementary file 1 [file sensors-18-00964-s001.pdf]

Supplementary Material

Green, hydrothermal synthesis of fluorescent carbon nanodots from gardenia enabling detection of metronidazole in pharmaceuticals and rabbit plasma

Xiupei Yang [1,]*, Mingxian Liu [1], Yanru Yin [1], Fenglin Tang [1], Hua Xu [1] and Xiangjun Liao [2]

[1] College of Chemistry and Chemical Engineering, Chemical Synthesis and Pollution Control Key Laboratory of Sichuan Province, China West Normal University, Nanchong 637000, China; mxliu_chem@163.com (M.L.); tfl06180205@126.com (F.T.); bigtree.xu@foxmail.com (H.X.)
[2] Exposure and Biomonitoring Division, Health Canada, 50 Colombine Driveway, Ottawa, K1A 0K9 Canada; xiangjun.liao@mail.mcgill.ca
* Correspondence: xiupeiyang@163.com; Tel.: +86-817-2568-081

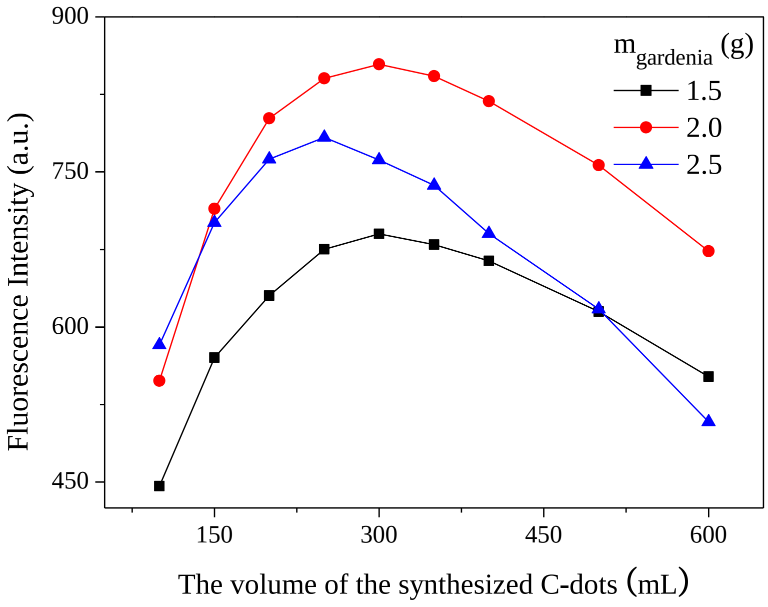

Figure S1. Effect of various quantity of gardenia for synthesis FCNs and differential dilution ratio on the fluorescence intensity of FCNs solution at 220 ℃ for 10 h.

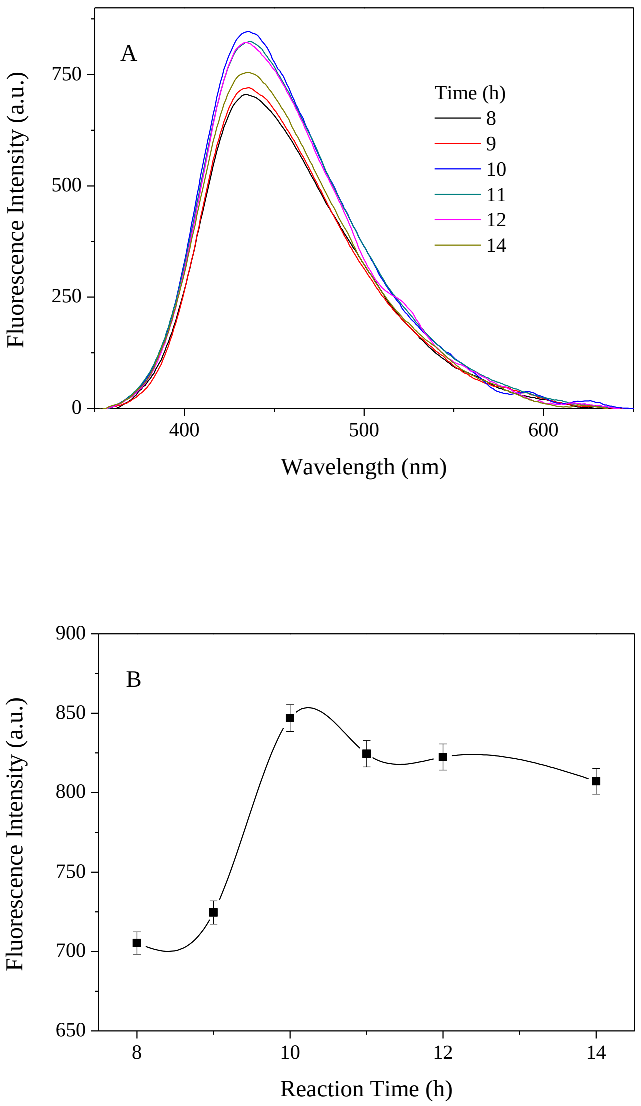

Figure S2. Fluorescence spectra (A) and fluorescence intensity (B) of C-dots preprared under various reaction times.

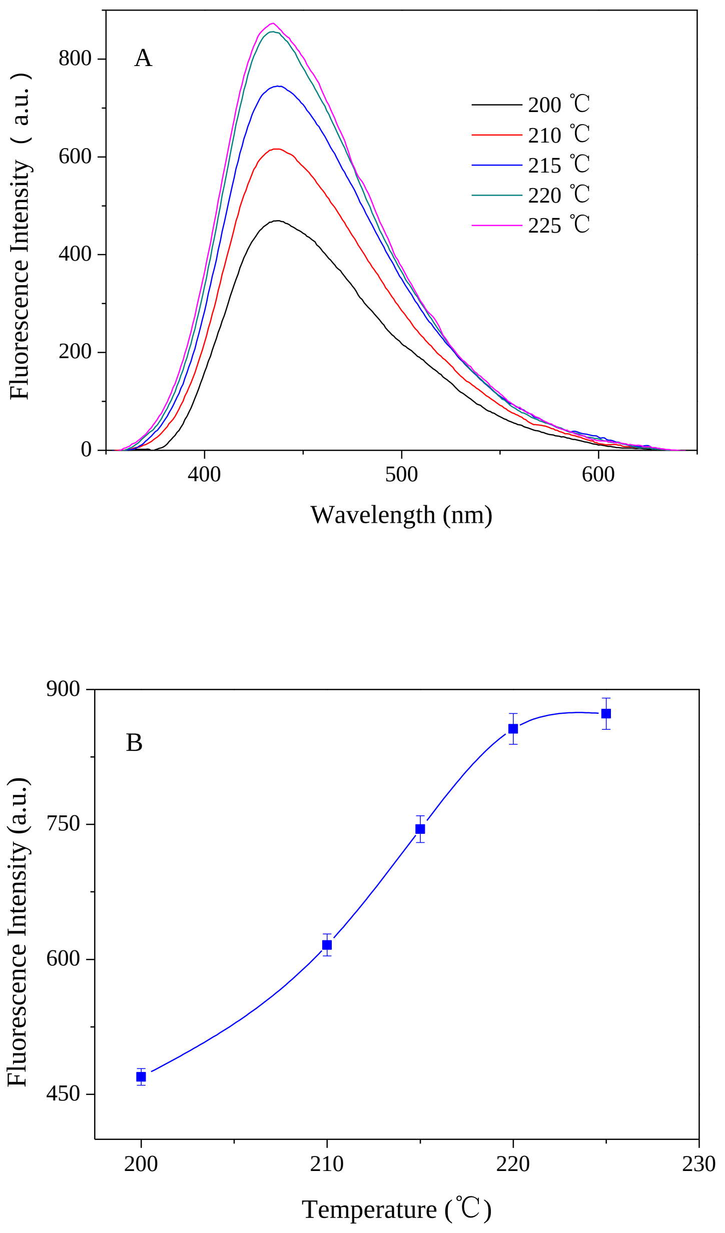

Figure S3. Fluorescence spectra (A) and fluorescence intensity (B) of C-dots preprared under various temperature.

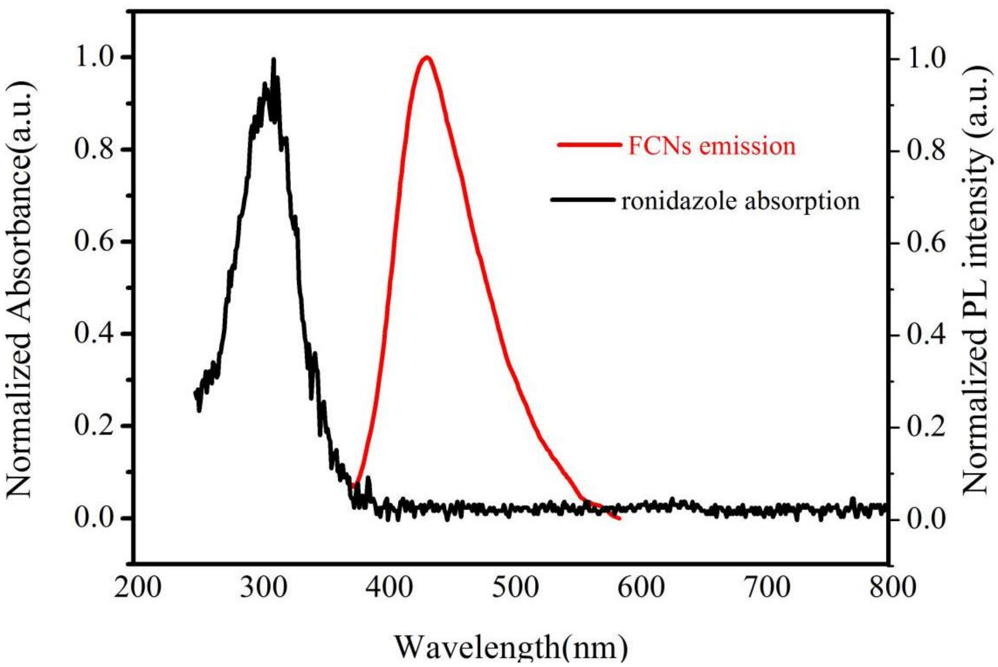

Figure S4. Overlapping between Flurescence spetra of FCNs and the UV-vis absorption spectra of ronidazole.

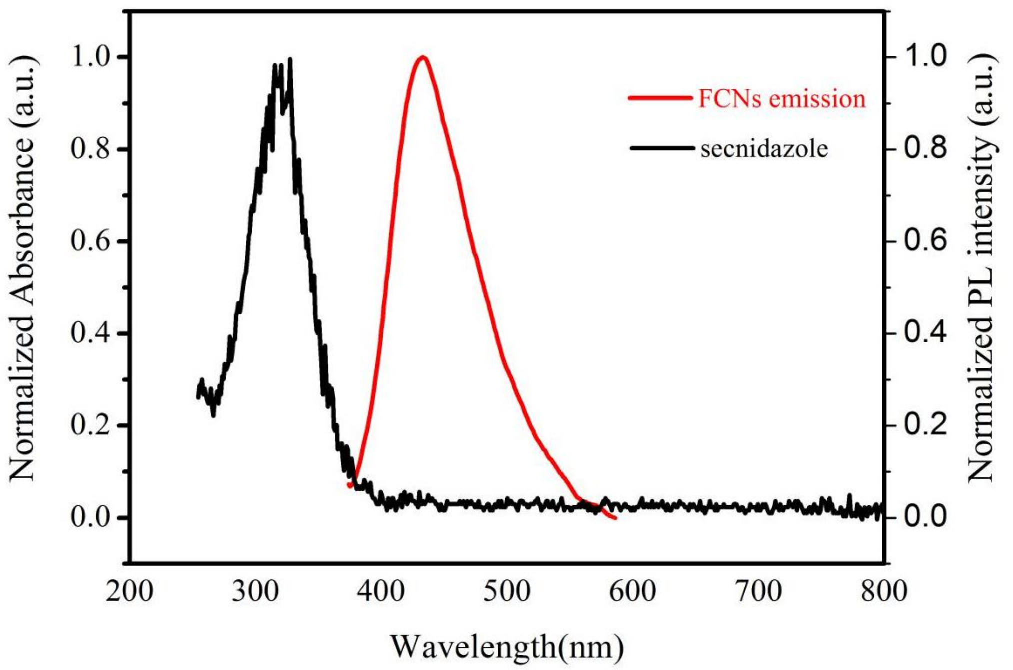

Figure S5. Overlapping between Flurescence spetra of FCNs and the UV-vis absorption spectra of secnidazole.

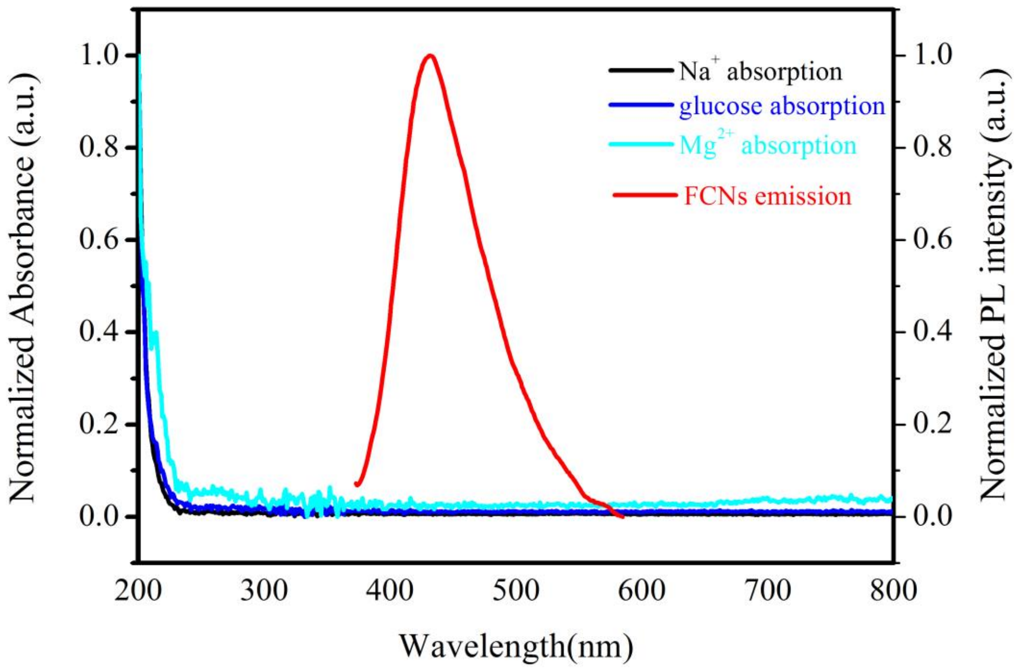

Figure S6. Overlapping between Flurescence spetra of FCNs and the UV-vis absorption spectra of glucose, Na^{+} and Mg^{2+}.

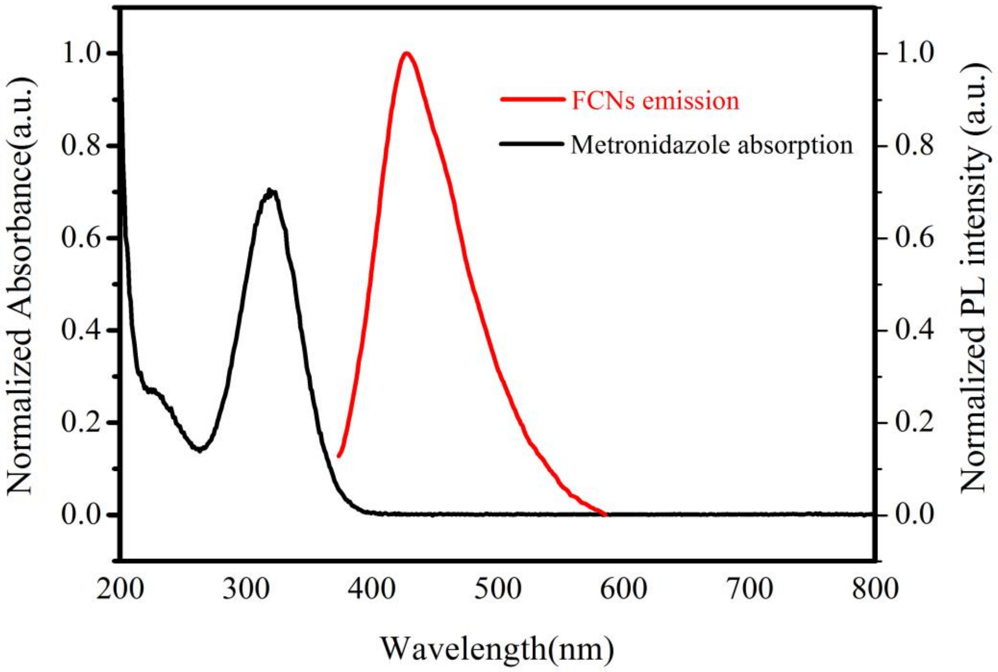

Figure S7. Overlapping between flurescence spetra of FCNs and the UV-vis absorption spectra of metronidazole.
